# Transposable elements in individual genotypes of *Drosophila simulans*

**DOI:** 10.1002/ece3.6134

**Published:** 2020-03-13

**Authors:** Sarah Signor

**Affiliations:** ^1^ Department of Biological Sciences North Dakota State University Fargo ND USA

**Keywords:** copy number, *Drosophila simulans*, genetic variation, inbred lines, transposable elements

## Abstract

Transposable elements are abundant, dynamic components of the genome that affect organismal phenotypes and fitness. In *Drosophila melanogaster*, they have increased in abundance as the species spread out of Africa, and different populations differ in their transposable element content. However, very little is currently known about how transposable elements differ between individual genotypes, and how that relates to the population dynamics of transposable elements overall. The sister species of *D. melanogaster*, *D. simulans*, has also recently become cosmopolitan, and panels of inbred genotypes exist from cosmopolitan and African flies. Therefore, we can determine whether the differences in colonizing populations are repeated in *D. simulans*, what the dynamics of transposable elements are in individual genotypes, and how that compares to wild flies. After estimating copy number in cosmopolitan and African *D. simulans*, I find that transposable element load is higher in flies from cosmopolitan populations. In addition, transposable element load varies considerably between populations, between genotypes, but not overall between wild and inbred lines. Certain genotypes either contain active transposable elements or are more permissive of transposition and accumulate copies of particular transposable elements. Overall, it is important to quantify genotype‐specific transposable element dynamics as well as population averages to understand the dynamics of transposable element accumulation over time.

## Graphical abstract

As species colonize new habitats, increased stress could lead to increased transposable element genome content. In Drosophila, cosmopolitan populations of *D. simulans* have higher transposable element copy number than African populations. However, while there is an overall difference in copy number, different transposable elements are active in each population, in different genotypes, and in *D. simulans* compared to *D. melanogaster*.

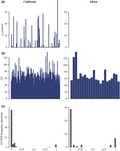

## 1 INTRODUCTION

Transposable elements are abundant, naturally occurring sources of genetic variation in populations, influencing genome evolution in diverse ways (Bennetzen, 2000; Biémont, Vieira, Borie, & Lepetit, 1999; Feschotte, 2008). Transposable elements can contribute to variation in quantitative traits, differences in fitness, and changes in gene expression (Mackay 1984; Mackay 1989; Shrimpton, Mackay, & Brown, 1990, Mackay, Lyman, Jackson, 1992, Long, Lyman, Morgan, Langley, & Mackay, 2000). Overall, due to the propensity of transposable elements to cause mutations, transposable elements are thought to be deleterious (Adrion, Song, Schrider, Hahn, & Schaack, 2017; Dimitri, 2003; Lee & Langley, 2012; Yang & Nuzhdin, 2003). However, transposable elements have also been associated with increases in fitness due to changes in gene regulation, where they can act as enhancers, repressors, or other regulators of complex gene expression patterns (Mateo, Ullastres, & González, 2014). The number and location of transposable element insertions can vary substantially between species, populations, and individuals (Jakšić, Kofler, & Schlötterer, 2017; Kofler, Nolte, & Schlötterer, 2015b; Kofler & Schlötterer, 2015; Kofler, Senti, Nolte, Tobler, & Schlötterer, 2018; Vieira, 2008; Vieira & Biémont, 2004; Vieira, Lepetit, Dumont, & Biémont, 1999).

*Drosophila melanogaster* has the most well‐annotated population of transposable elements. Transposable elements are active in *D. melanogaster*, with insertion rates between 10^−3^ and 10^−5^ elements per generation (Nuzhdin and Mackay 1994). Most transposable element insertions segregate at low population frequencies, due to either selection against insertions (transposition selection balance) or recent bursts of transposable element activity (Blumenstiel, Chen, He, & Genetics, 2014; Kofler, Nolte, et al., 2015). Newly invading transposable elements may initially have high transposition rates as the host machinery evolves new defenses (Johnson, 2010; Lee & Langley, 2012; Pasyukova, 2004; Romero‐Soriano & Garcia Guerreiro, 2016; Slotkin & Martienssen, 2007; Yang & Nuzhdin, 2003).

*Drosophila* *melanogaster* recently evolved to be a human commensal and spread out of Africa to a worldwide distribution (around 10,000 years ago (Baudry, 2004; Kauer, Zangerl, Dieringer, & Schlötterer, 2002; Sprengelmeyer et al., 2018; Wu et al., 1995; Yukilevich, Turner, Aoki, Nuzhdin, & True, 2010)). When organisms colonize new habitats, conditions may be stressful and they may encounter new congeners. Both of these conditions could potentially result in an increase in transposable element activity, through introgression and reduced efficacy of the organism's system for repressing transposable element activity, such as piRNA (Engels, 1992; Kofler, Nolte, et al., 2015). *D. melanogaster* from Africa have been observed to have a lower number of transposable element insertions than cosmopolitan *D. melanogaster*, which has been attributed to a “waking up” of transposable elements upon colonization of new habitats (Vieira et al., 1999). The sister species of *D. melanogaster, D. simulans,* also originated in Afrotropical climates and evolved into human commensals with cosmopolitan distributions throughout Europe and the Americas, albeit more recently (Sturtevant, 1920). Due to its more recent spread, and heterogeneity among populations in their transposable element content, it was previously proposed that the waking up of transposable elements in *D. simulans* is currently in progress (Vieira et al., 1999).

More recently, the most frequently used approach to studying transposable element abundance in *D. melanogaster* and *D. simulans* has been Pool‐seq. Pool‐seq has generated some interesting observations about transposable element dynamics; for example, in *D. melanogaster* it has confirmed that transposable elements are more abundant in cosmopolitan populations than in their ancestral African range (Kofler, Nolte, et al., 2015). Pool‐seq documented the recent invasion of the P‐element into *D.* *simulans* from *D. melanogaster*, highlighting the ever‐changing transposable element landscape between species and populations (Kofler, Hill, Nolte, Betancourt, & Schlötterer, 2015a). While Pool‐seq may an effective tool for estimating population‐level frequency, there is evidence that estimates of transposable element insertion dynamics can be confounded by differences in allele frequencies (Rahman et al. 2015). Furthermore, it is informative to estimate the variance between genotypes in transposable element copy number, in addition to population‐level variation. For example, how much of the observed population‐level variation is due to individuals with high copy number rather than low population averages?

In *D. melanogaster*, the existence of multiple sequenced inbred panels lends themselves to estimating copy number and insertion site frequency between individual genotypes. Active families of transposable elements appear to be largely shared between populations, for example, in inbred strains of *D. melanogaster* from worldwide samples, the DGRP, and pooled noninbred flies from global samples; the majority of transposable element insertions are from the same six transposable element families (Rahman et al. 2015). However, these estimates of specific differences in transposable element load between genotypes were performed on a limited number of strains and have not been performed in other systems, including in *D. simulans*.

Here, I will specifically address three of these questions in *D. simulans,* to understand what observations from *D. melanogaster* are unique to the species and which are shared*.* First, how do transposon families differ between fly genotypes and which transposon families are most prevalent in these differences? Second, how do transposable elements differ between cosmopolitan and ancestral *D. simulans*? Third, how much difference do we see between *D. simulans* sequenced from inbred lines versus those sequenced directly from wild collections? I estimate variance in transposable element copy number between inbred genotypes, differences between wild and inbred lines, and differences between the populations in the mean and variance of transposable element copy number.

## 2 METHODS

### 2.1 Fly lines

Twenty‐one African *D. simulans* isofemale lines were collected by William Ballard in 2002 from Madagascar and Peter Andolfatto in 2006 from Kenya (Table 1, Jackson, Campos, Haddrill, Charlesworth, & Zeng, 2017). They were inbred in the laboratory for nine generations. During the process of inbreeding, five were lost and were sequenced from the original wild sample which had been preserved in ETOH (Table 1). These five lines will be used as an estimate of “wild” *Drosophila* transposable element load, compared to the inbred lines. The raw reads are 90‐bp paired‐end Illumina sequencing, and they were downloaded from SRA PRJEB7673 (Jackson et al., 2017). The first read from each pair was used for mapping. The 169 California lines were collected from the Zuma Organic Orchard in Los Angeles, CA, on two consecutive weekends of February 2012 (Table 1; Signor, New, & Nuzhdin, 2017; Signor & Nuzhdin, 2018, 2019). Reads were single‐end 100 bp, and this project has been deposited at the SRA under accession SRP075682.

**Table 1:** A list of the strains used for this study, including their collection location and inbreeding status

	African *Drosophila*		Cosmopolitan *Drosophila*
Collection	Kenya	Madagascar	California
N. Strains	11	10	169
N. Inbred	8	8	169

### 2.2 Mapping and copy number estimation

**Figure 1:**
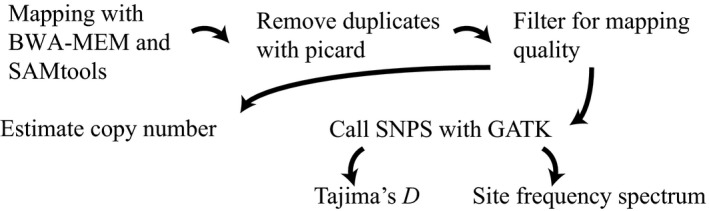
An outline of the pipeline used for estimating copy number of transposable elements in *Drosophila* *simulans*, as well as estimating the site frequency spectrum

Example scripts for all of the following methods are available at https://github.com/signor-molevol/simulans_transposable. Reads were mapped using BWA‐MEM version 0.7.15 to the *D. simulans* 2.02 assembly and the 179 consensus transposable element sequences from EMBL, downloaded from Flybase.org (Figure 1; Li, 2015, reference also available at https://github.com/signor-molevol/simulans_transposable). Of these, 128 were used for the analysis, removing those from non‐*D. melanogaster* species that did not have a presence in *D. simulans*. Bam files were sorted and indexed with SAMtools v.1.9, and optical duplicates were removed using picard MarkDuplicates (http://picard.sourceforge.net) (Li et al., 2009; McKenna et al., 2010). Reads with a mapping quality of below 15 were removed (this removes reads which map equally well to more than one location). Using read coverage to determine copy number has been compared to other methods and is neither permissive nor conservative (Srivastav & Kelleher, 2017). Transposable element copy number was estimated per family by estimating the average counts of reads mapping to the transposable element sequences and the genome with bedtools counts (Hill, Schlötterer, & Betancourt, 2015; Quinlan & Hall, 2010). Then, copy number of the transposable elements could be normalized using the average counts from 2 L in R. Significance of the difference between populations was determined using a *t* test for means and an *F* test for variance. *p*‐values of comparisons between means and variances were corrected for multiple testing using Bonferroni correction.

### 2.3 SNPs and summary statistics

I called SNPs within the consensus sequence of the transposable elements and the genomes using GATK Haplotypecaller (McKenna et al., 2010). SNPs were filtered for a minimum depth of four. SNPs were not filtered for missing calls given that not at all individuals will share insertions. Tajima's *D* was estimated in windows of 1 kb using VCFtools, and prior to estimation indels and SNPs with more than two alleles were removed (Danecek et al., 2011). The site frequency spectrum of SNPs was estimated with VCFtools as the frequency of each SNP in the population, and then the frequency of the SNP frequencies was estimated in R (Danecek et al., 2011).

## 3 RESULTS AND DISCUSSION

### 3.1 Population‐level variation

Of the 128 elements examined in the population, 85 have different mean numbers of insertions between the two populations (*t* test, Bonferroni‐corrected *p* = .05/128, Table 2, Table S1). Of those, only 17 are higher in the African populations, suggesting that overall the CA population has more transposable element insertion sites. Indeed, overall Californian *D. simulans* have an average of 1,797 insertions per genotype, while African *D. simulans* have 1,496 (Table 2). The five elements with the largest difference in copy number in Californian *D. simulans* compared to African are the *INE‐1*, *Tc1*, *transib2*, *1,360*, and *Cr1a* (Table 2). These are present on average in 37 more copies in Californian *D. simulans*. Fifteen elements also have significantly different (and higher) variance in African *D. simulans* compared to Californian *D. simulans* (*F* test, Bonferroni‐corrected *p* = .05/128). Twenty elements have different and larger variance in the Californian *D. simulans* compared to African (*F* test, Bonferroni‐corrected *p* = .05/128). The most abundant transposable elements in each population tend to be abundant in both populations, namely *INE‐1*, *Cr1a*, and *G6*. The *D. melanogaster pogo* and *Helitron* elements were not present in these populations, which has been previously noted, suggesting that these transposable elements are not present in *D. simulans* (Kofler, Nolte, et al., 2015). Previous work using Pool‐seq in *D. simulans* identified *INE‐1*, *roo*, *Cr1a*, *Rt1c*, and *hobo* as the most abundant transposable elements in *D. simulans*, and *G6* was among the less abundant elements (Kofler, Nolte, et al., 2015).

**Table 2:** Summary statistics for copy number of transposable elements from the Californian (CA) and African (AF) *Drosophila* *simulans*

TE family	CA average	Variance	AF average	Variance	TE family	CA average	Variance	AF average	Variance	TE family	CA average	Variance	AF average	Variance
*297*	18.44	7.87	12.11	8.31	*GATE*	26.98	12.73	28.32	13.07	*NOF*	1.04	0.21	0.97	0.32
*412*	27.78	31.66	14.77	5.39	*gtwin*	4.85	0.73	4.78	0.87	*opus*	7.64	5.03	6.85	9.54
*1,360*	64.03	37.76	35.07	20.84	*gypsy*	4.97	1.06	5.92	2.02	*Osvaldo*	5.44	0.85	6.96	1.38
*1731*	6.69	6.86	7.10	3.65	*gypsy10*	8.37	5.13	6.34	3.94	*P‐element*	2.36	44.92	‐	‐
*17.6.*	1.10	0.13	0.50	0.00	*gypsy11*	3.73	0.29	2.42	0.08	*pogo*	‐	‐	‐	‐
*3S18*	11.89	5.66	12.59	4.38	*gypsy12*	3.97	0.75	4.51	0.86	*Porto1*	10.02	0.65	7.49	0.39
*accord*	6.05	10.35	2.54	1.17	*gypsy2*	0.95	0.02	0.81	0.01	*Q‐element*	0.95	0.03	0.72	0.01
*accord2*	4.84	0.81	3.23	0.41	*gypsy3*	3.45	0.95	3.08	0.73	*Quasimodo*	0.19	0.03	0.08	0.00
*aurora‐element*	0.70	0.02	0.89	0.02	*gypsy4*	2.07	0.04	1.98	0.08	*R1‐2*	1.13	0.05	1.21	0.11
*baggins*	52.65	15.51	62.04	42.68	*gypsy5*	3.68	1.12	2.26	0.52	*R1A1‐element*	34.07	72.24	49.39	301.81
*Bari1*	4.92	4.58	18.05	30.07	*gypsy6*	0.39	0.01	0.31	0.00	*R2‐element*	39.52	65.11	30.05	74.21
*Bari2*	0.12	0.00	0.11	0.00	*gypsy7*	0.86	0.01	0.64	0.00	*roo*	50.89	66.75	33.38	43.96
*blood*	12.12	11.30	7.98	10.57	*gypsy8*	4.77	0.69	6.38	1.95	*rooA*	19.29	1.30	18.61	1.19
*BS*	2.93	0.27	2.94	0.17	*gypsy9*	3.14	0.24	2.46	0.23	*rover*	1.24	0.15	0.58	0.03
*BS3*	6.41	0.63	6.95	0.51	*HB*	43.04	20.11	40.52	32.24	*Rt1a*	3.61	0.26	3.91	0.38
*BS4*	3.31	0.13	2.93	0.07	*Helena*	29.20	17.08	22.24	10.19	*Rt1b*	17.43	6.41	24.71	8.67
*Burdock*	12.73	4.35	9.27	3.04	*Helitron*	‐	‐	‐	‐	*Rt1c*	20.60	14.16	27.78	41.37
*Circe*	6.56	1.79	5.88	2.80	*HeT‐A*	6.52	4.70	1.50	1.59	*S‐element*	1.41	0.06	0.83	0.05
*copia*	9.22	4.00	6.52	11.85	*HMS‐Beagle*	13.44	2.59	10.49	2.17	*S2*	0.73	0.01	0.57	0.00
*Cr1a*	125.69	88.15	97.02	39.36	*HMS‐Beagle2*	0.82	0.01	0.69	0.00	*springer*	2.10	0.10	2.11	0.09
*diver*	6.75	8.19	8.14	26.95	*hobo*	32.87	12.41	31.79	32.23	*Stalker*	0.28	0.00	0.19	0.00
*diver2*	35.58	21.62	44.05	32.27	*hopper*	23.58	16.70	15.88	11.24	*Stalker2*	2.86	3.49	2.31	1.14
*Dm88*	8.43	0.45	8.34	0.34	*hopper2*	0.77	0.06	0.66	0.05	*Stalker3*	0.08	0.03	‐	‐
*Dmau\mariner*	1.22	0.72	2.33	1.54	*I‐element*	37.33	35.49	21.37	13.78	*Stalker4*	0.09	0.00	0.06	0.00
*Doc*	25.20	24.77	23.59	37.24	*Idefix*	3.70	1.91	2.64	0.57	*Tabor*	9.95	44.94	5.81	3.25
*Doc2‐element*	30.43	6.53	28.56	8.46	*INE‐1*	193.45	296.57	116.27	104.78	*TAHRE*	3.09	1.10	1.01	1.35
*Doc3‐element*	20.57	1.17	19.21	1.48	*invader1*	4.62	0.11	4.55	0.18	*TART‐A*	1.05	0.24	0.54	0.09
*Doc4‐element*	3.68	0.25	2.99	0.07	*invader2*	6.09	1.50	4.29	0.92	*TART‐B*	0.94	0.20	0.36	0.15
*Dsim\ninja*	3.14	4.99	4.93	5.85	*invader3*	6.71	1.93	6.47	5.80	*TART‐C*	0.02	0.00	0.01	0.00
*F‐element*	28.41	11.23	19.78	1.48	*invader4*	3.24	0.08	3.07	0.06	*Tc1*	54.48	36.25	31.21	10.81
*FB*	22.44	20.74	13.75	14.15	*invader5*	0.66	0.01	0.56	0.00	*Tc1‐2*	12.45	2.47	8.46	1.85
*flea*	13.71	10.09	10.85	13.78	*invader6*	5.85	1.55	6.20	3.67	*Tc3*	0.15	0.00	0.06	0.00
*frogger*	1.05	0.06	0.89	0.01	*Ivk*	14.98	2.26	12.87	2.72	*Tirant*	2.77	0.06	3.58	2.17
*Fw2*	6.50	0.20	5.64	0.14	*jockey*	8.73	3.10	11.61	4.33	*Tom1*	26.03	6.41	17.00	7.94
*Fw3*	3.01	0.07	2.89	0.16	*jockey2*	5.55	0.15	4.68	0.04	*transib1*	19.12	8.84	12.41	4.99
*G‐element*	4.22	0.93	4.08	1.26	*Juan*	21.46	27.13	18.68	13.75	*transib2*	55.12	44.06	27.48	39.43
*G2*	25.99	15.12	23.93	16.78	*looper1*	2.24	0.11	1.56	0.07	*transib3*	2.81	0.07	2.07	0.07
*G3*	0.91	0.01	0.76	0.01	*mariner2*	9.61	0.54	7.34	0.28	*transib4*	4.14	0.17	2.80	0.16
*G4*	4.10	0.23	3.52	0.06	*Max‐element*	29.78	17.50	32.43	15.97	*Transpac*	8.21	4.81	5.35	4.09
*G5*	15.05	2.32	15.99	2.77	*McClintock*	13.49	8.00	6.86	2.81	*X‐element*	34.10	8.98	41.49	23.77
*G5A*	10.93	0.66	11.67	2.70	*mdg1*	6.43	1.00	4.95	0.85	*Xanthias*	9.43	3.14	8.90	6.90
*G6*	66.62	309.64	69.36	532.88	*mdg3*	7.02	4.73	5.94	3.01	*ZAM*	2.92	0.12	2.01	0.23
*G7*	0.18	0.00	0.20	0.01	*micropia*	8.01	4.05	6.53	2.71					

Some elements are not present in full‐length copies within either population. Six transposable elements (*Stalker4, Stalker*, *Bari2*, *Tc3*, *G7*, and *Tart‐C*) were never present as more than a fraction of an element in any individual, and they are likely old and degraded. *G3* and *hopper2* are estimated as being present in ~1 copy per individual in both populations; however, that copy or copies has internal deletions. For the *G‐element,* all but a small fraction of reads map to one 140 bp sequence. A full‐length version of *Quasimodo* (two copies) and *gypsy6* (one copy) were present in one genotype, while in other genotypes *Quasimodo* appears to be old and degraded. *Stalker3* is also present in one genotype as a full‐length copy; however in this case, old or degraded copies are not present in the other genotypes. Reads which map equally well to more than one location were filtered out; thus, this does not represent nonspecific mapping to repetitive elements.

### 3.2 Site frequency spectrum

I examined the site frequency spectrum of each transposable element in African and Californian *D. simulans* (Table 3). In some cases, there are no polymorphisms (*Dmau\mariner, Dmel\p‐element*); therefore, this is uninformative. Genome‐wide, the Californian population has more intermediate frequency polymorphisms (measured using Tajima's *D*; Signor et al., 2017) compared to the African population (Figure S1), which may be expected to affect the site frequency spectrum. The site frequency spectrum must be interpreted along with Table 2—for example, *Quasimodo* is really only present in two full‐length copies in a single individual; thus, this estimation of the site frequency spectrum is not informative with regard to the spread of *Quasimodo* in the population.

**Table 3:** Average site frequency spectrum for each element, excluding sites that are fixed relative to *Drosophila* *melanogaster*

TE family	CA average	AF average	TE family	CA average	AF average	TE family	CA average	AF average
*297*	0.29	0.29	*GATE*	0.28	0.53	*NOF*	0.37	0.37
*412*	0.31	0.31	*gtwin*	0.35	0.35	*opus*	0.38	0.38
*1,360*	0.26	0.26	*gypsy*	0.35	0.35	*Osvaldo*	0.37	0.37
*1731*	0.23	0.23	*gypsy10*	0.35	0.35	*P‐element*	‐	‐
*17.6.*	0.44	0.44	*gypsy11*	0.44	0.44	*pogo*	‐	‐
*3S18*	0.16	0.16	*gypsy12*	0.35	0.35	*Porto1*	0.41	0.41
*accord*	0.08	0.08	*gypsy2*	0.42	0.42	*Q‐element*	0.32	0.32
*accord2*	0.36	0.36	*gypsy3*	0.33	0.33	*Quasimodo*	0.05	0.05
*aurora‐element*	0.31	0.31	*gypsy4*	0.22	0.22	*R1‐2*	0.33	0.33
*baggins*	0.29	0.29	*gypsy5*	0.24	0.24	*R1A1‐element*	0.32	0.32
*Bari1*	0.05	0.05	*gypsy6*	0.33	0.33	*R2‐element*	0.35	0.35
*Bari2*	0.40	0.40	*gypsy7*	0.24	0.24	*roo*	0.14	0.14
*blood*	0.18	0.18	*gypsy8*	0.36	0.36	*rooA*	0.32	0.32
*BS*	0.34	0.34	*gypsy9*	0.41	0.41	*rover*	0.42	0.42
*BS3*	0.42	0.42	*HB*	0.34	0.34	*Rt1a*	0.39	0.39
*BS4*	0.37	0.37	*Helena*	0.19	0.19	*Rt1b*	0.29	0.29
*Burdock*	0.22	0.22	*Helitron*	‐	‐	*Rt1c*	0.35	0.35
*Circe*	0.28	0.28	*HeT‐A*	0.43	0.43	*S‐element*	0.42	0.43
*copia*	0.11	0.11	*HMS‐Beagle*	0.32	0.32	*S2*	0.42	0.42
*Cr1a*	0.24	0.24	*HMS‐Beagle2*	0.42	0.42	*springer*	0.35	0.35
*diver*	0.29	0.29	*hobo*	0.23	0.23	*Stalker*	0.28	0.28
*diver2*	0.28	0.28	*hopper*	0.33	0.33	*Stalker2*	0.15	0.15
*Dm88*	0.40	0.40	*hopper2*	0.37	0.37	*Stalker3*	‐	‐
*Dmau\mariner*	‐	‐	*I‐element*	0.26	0.26	*Stalker4*	0.25	0.25
*Doc*	0.15	0.15	*Idefix*	0.38	3.70	*Tabor*	0.08	0.08
*Doc2‐element*	0.20	0.20	*INE‐1*	0.31	0.31	*TAHRE*	0.40	0.40
*Doc3‐element*	0.35	0.35	*invader1*	0.39	0.39	*TART‐A*	0.31	0.31
*Doc4‐element*	0.37	0.37	*invader2*	0.29	0.29	*TART‐B*	0.34	0.34
*Dsim\ninja*	0.30	0.30	*invader3*	0.20	0.20	*TART‐C*	0.10	0.10
*F‐element*	0.22	0.22	*invader4*	0.40	0.40	*Tc1*	0.34	0.34
*FB*	0.37	0.37	*invader5*	0.44	0.44	*Tc1‐2*	0.40	0.40
*flea*	0.04	0.04	*invader6*	0.14	0.14	*Tc3*	0.08	0.08
*frogger*	0.17	0.17	*Ivk*	0.34	0.34	*Tirant*	0.35	0.35
*Fw2*	0.44	0.44	*jockey*	0.10	0.10	*Tom1*	0.29	0.29
*Fw3*	0.36	0.36	*jockey2*	0.40	0.40	*transib1*	0.30	0.30
*G‐element*	0.53	0.53	*Juan*	0.06	0.06	*transib2*	0.34	0.34
*G2*	0.35	0.35	*looper1*	0.34	0.34	*transib3*	0.37	0.37
*G3*	0.50	0.50	*mariner2*	0.42	0.42	*transib4*	0.40	0.40
*G4*	0.33	0.33	*Max‐element*	0.39	0.39	*Transpac*	0.04	0.04
*G5*	0.34	0.34	*McClintock*	0.19	0.19	*X‐element*	0.25	0.27
*G5A*	0.37	0.37	*mdg1*	0.40	0.40	*Xanthias*	0.25	0.25
*G6*	0.10	0.10	*mdg3*	0.15	0.15	*ZAM*	0.31	0.31
*G7*	0.36	0.36	*micropia*	0.24	0.24			

Note

*Dmau\mariner* and the *p‐element* are present in at least one population, but have no polymorphic SNPs. Other elements without an estimated site frequency spectrum are not present in the population.

**Figure 2:**
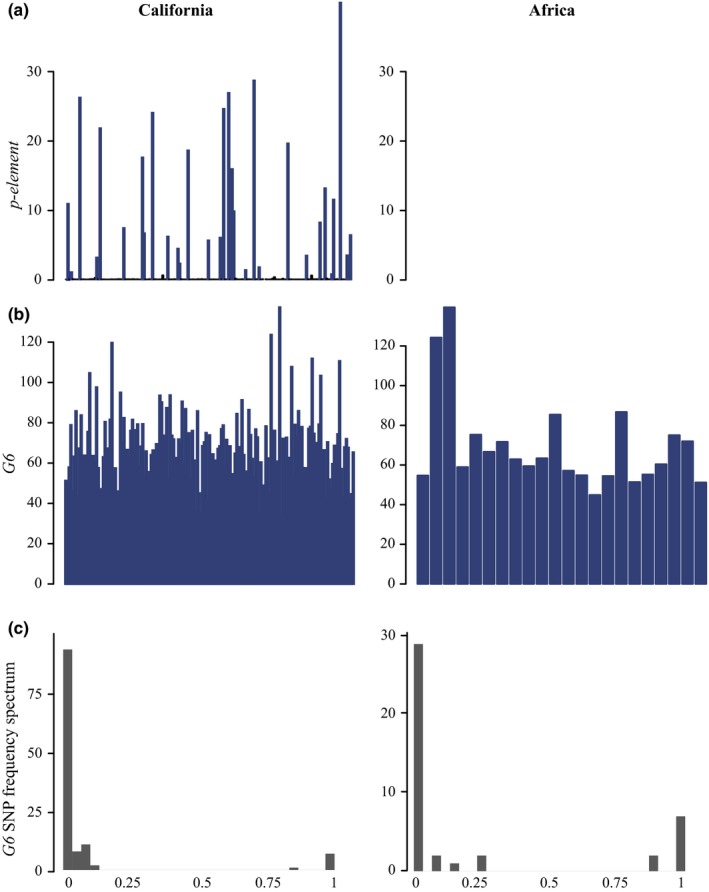
(a) Estimated copy number for the *p‐element* in Californian and African *Drosophila* *simulans*. Each bar represents an individual from the population. As expected, the *p‐element* was not found in the African population sampled in the early 2000s, but by 2012 when the Californian *D. simulans* was sampled, it had invaded. (b) Estimated copy number for *G6* in Californian and African *D. simulans*. Each bar represents an individual from the population *G6* has a high copy number in both populations of *D. simulans*, which was not recorded in previous studies on African *D. simulans*. (c) The site frequency spectrum in the last row also suggests recent spread of the *G6* element in *D. simulans*, as there are primarily low‐frequency SNPs. Note that while fixed SNPs are included in this graph to illustrate divergence from *Drosophila* *melanogaster,* they are not included in the estimation of average site frequency spectrum shown in Table 3

Elements with site frequency spectrum heavily biased toward low‐frequency SNPs in Californian *D. simulans* include *G6*, *flea*, and *Juan* (Figure 2, Table 3). In African *D. simulans,* this includes *Tabor*, *Transpac*, *flea*, *Juan*, *Bari1*, *G6*, and *accord* (Figure 2, Table 3)*.* Thus in both populations, *G6*, *flea*, *Bari1*, and *Juan* likely have recent activity. This is consistent with other work on *Juan*, which suggests it is actively transposing in the species (Kofler, Nolte, et al., 2015). The larger number of transposable elements with low‐frequency SNPs in African populations may be due to the overall difference in the site frequency spectrum between populations (Figure S1; Signor et al., 2017).

### 3.3 The p‐element

The *p‐element* recently invaded *D. simulans* from *D. melanogaster* as described in Kofler, Hill, et al. (2015); however, Pool‐seq cannot tie *p‐element* insertions to specific individuals and only determine the average number of insertions. What was reported previously was 0.4 insertions in Florida populations and 29 in South Africa (Kofler, Hill, et al., 2015). What we see in the California population is an average of two insertions, however that is because the majority of individuals do not have any insertions (137 individuals have less than 0.3 estimated copies, Figure 2). The remaining individuals have between 0.5 and 39 copies. It is interesting that it is not invading genotypes in the population at the same rate, but rather reaching high copy number in some genotypes and not others (Nuzhdin, 2000). It is possible that *p‐elements* are just proliferating in strains that contained an active copy prior to collection (Nuzhdin, Pasyukova, & Mackay, 1997). This was observed previously in laboratory strains of *D. melanogaster*, though contamination and introgression may also have played a role (Rahman et al. 2015).

### 3.4 Transposable elements in individual genotypes

**Figure 3:**
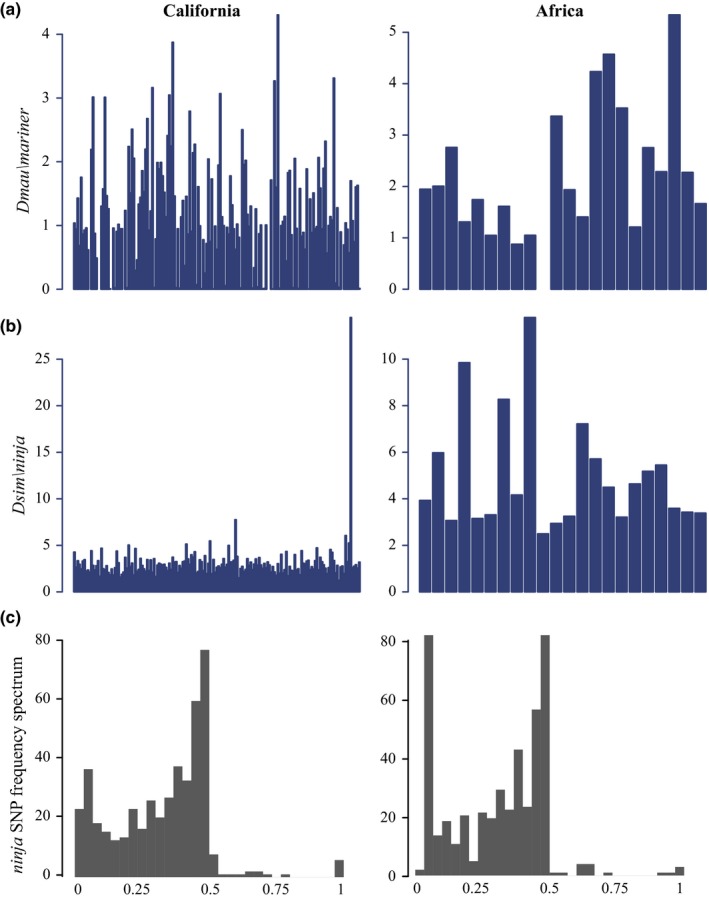
(a,b) Estimated copy number for the two non‐*Drosophila* *melanogaster* transposable elements included here, *Dsim\ninja* and *Dmau\mariner*. (c) The site frequency spectrum of *Drosophila* *sim\ninja* for Californian and Africa *D. simulans*. The site frequency spectrum of *D. sim\ninja* is broad, suggesting that outside of the genotype with an active copy of *D. sim\ninja* this element has been diverging within this species for some time. In contrast, no SNPs were called in *Dmau\mariner*, suggesting recent colonization in *D. simulans*

Some transposable elements have considerably higher copy number in particular genotypes compared to the population average. For example, in one genotype *Dsim\ninja* is present in 29 copies, compared to the population mean of three (Figure 3). *Dsim\ninja* has 10 fixed differences and 27 polymorphisms in this strain from the California population, and the population average is 7.5 fixed differences and 264 polymorphisms. This suggests that *Dsim\ninja* was recently active in this genotype. This is true of several transposable elements which have outliers in the population. *Stalker2* has an outlier genotype with 17 fixed SNPs and eight polymorphisms, compared to a population average of 14 fixed SNPs and 43 polymorphisms. Other transposable elements with large outliers in the California population include *gypsy10*, *opus*, *blood*, *GATE*, *diver*, *Tabor*, *INE‐1*, *diver2*, *idefix*, *1731*, *412*, and *297*.

Sampling of the African populations was much more limited; thus, less genotype‐specific variation is sampled, and indeed, only two transposable elements had large outliers, in both the same genotype from Madagascar: *copia* and *diver*. This genotype had 20 copies of *copia*, compared to a population frequency of 4–11, as well as 11 fixed differences and 20 polymorphisms (compared to a population average of 10 fixed differences and 78 polymorphisms). For *diver,* this genotype had 20 fixed differences and 84 polymorphisms, compared to a population average of 12 fixed differences and 215 polymorphisms (and 30 copies compared to 4–10 for the rest of the population).

### 3.5 *Wild *versus* inbred strains of D. simulans*

The outlier genotype from Africa that has more copies of *copia* and *diver* is one that was inbred in the laboratory. In general, being inbred in the laboratory is not affecting overall transposable element copy number; however, as comparing between lines that were sequenced directly upon collection and those that there inbred, there is no significant difference between the mean number of transposable elements for any transposable element family. The activity of *copia* and *diver* is specific to a genotype, rather than to “wild” or “inbred” strains. Those that “wake up” in individual lines appears to be due to sampling of individuals that are permissive or contain active transposable elements, rather than an overall increase in transposable element activity in inbred lines.

### 3.6 Comparison to other studies

*Tirant* has previously been reported as having higher copy number in African *D. simulans*, potentially due to a recent mobilization of the element (Fablet, McDonald, Biémont, & Vieira, 2006). We find that pattern here, including a higher variance in the African populations where copy number ranges from 2 to 6.68, compared to 2–3.8 in California (Fablet et al., 2006). The *Dmau\mariner* element has a higher copy number in Africa than in the Californian *D. simulans*, from 0–5 with an average of 2.33, compared to 0–3 with an average of 1.22 (Figure 3). *Dmau\mariner* also contains no polymorphisms, which is consistent with a recent spread of *Dmau\mariner* in *D. simulans* (Capy, Chakrani, Lemeunier, Hartl, & David, 1990; Capy, Koga, David, & Hartl, 1992). The *G6* element has a large difference from previously reported values, with an average of 66 insertions in Californian *D. simulans* and 69 in African. However, only 37 insertions were reported total for a previously estimated population of ~800 *D. simulans* isofemale lines (Kofler, Nolte, et al., 2015). In addition, in the populations reported here the *G6* element has primarily low‐frequency polymorphisms (Table 3), suggesting that this is a recent expansion of copy number. Overall, our estimates are higher than the work of Kofler, Hill, et al., 2015, which focuses on euchromatic insertions and only estimates more than one insertion per line for four transposable elements (*1,360*, *hobo*, *roo,* and *Tc‐2*).

### 3.7 Comparison to D. melanogaster

*INE‐1*, *1,360*, *jockey*, *hobo*, *roo*, and *p‐element* have been estimated as the most abundant transposable elements in *D. melanogaster* (Rahman et al., 2015; Kofler, Nolte, et al., 2015). The most abundant transposable elements in both populations of *D. simulans* in this study were *G6*, *Cr1a*, and *INE‐1*. However, *roo* and *hobo* are both quite abundant in *D. simulans,* and *1,360* is abundant in cosmopolitan populations. In both *D. melanogaster* and *D. simulans*, transposable element copy number is lower in African populations, suggesting that colonization is associated with increased transposable element activity (Vieira et al., 1999). However, overall the lack of reporting of individual population values makes comparison difficult.

In *D. simulans,* there is some evidence, either genotypes with large increases in copy number or a site frequency spectrum biased toward low‐frequency alleles, that *Dsim\ninja*, *Dmau\mariner*, *p‐element*, *gypsy10*, *opus*, *blood*, *GATE*, *diver*, *Tabor*, *INE‐1*, *diver2*, *Idefix*, *1731*, *412*, *297, G6, flea, Bari1, Transpac, Tabor, accord,* and *Juan* are active*. gypsy10, blood, Juan, G6, Tabor, Transpac, accord*, and *diver* have been previously reported as undergoing a burst of activity in *D. simulans* and in *D. melanogaster*, likely due to recent invasion (Kofler, Nolte, et al., 2015). *Flea*, *Idefix*, *412*, and *297* are also thought to be active, though due to an older invasion in the genome of *D. melanogaster* (Kofler, Nolte, et al., 2015). *G6* has been reported as having low copy number in *D. melanogaster*; however, it was also potentially recently active. Thus, *D. melanogaster* and *D. simulans* share many active families of transposable elements and appear to be experiencing an increase in transposable element copy number concurrent with worldwide expansion.

## 4 CONCLUSIONS

*Drosophila* *simulans* is currently being invaded by transposable elements, and this spread is likely occurring concordant with the worldwide colonization of *D. simulans*, as has been posited by previous studies (Lachaise et al., 1988; Vieira et al., 1999; Biémont et al., 2003). African populations have their own transposable element dynamics, with some transposable elements seeming to share activity between populations (*G6*) and others being more active in African *D. simulans* (*baggins*, *Bari1*, etc.). It would be interesting to explore transposable element dynamics in other populations of *D. simulans* to understand the generality of the patterns seen here. Transposable element load is an attribute of species, populations, and individual genotypes. In inbred laboratory genotypes, active transposable element copies may be inherited by some genotypes and not others, and active transposable elements can accumulate over time (Nuzhdin et al., 1997). This can cause differences over time in the number of insertions within a genotype and large differences between genotypes in transposable element copy number (Nuzhdin et al., 1997). This may also be reflective of natural patterns in which transposable elements proliferate in particular genotypes rather than at low levels in the population as a whole (Nuzhdin, 2000). Overall, looking at variance between individuals is an important part of understanding the ways in which transposable elements maintain themselves in populations.

## CONFLICT OF INTEREST

None declared.

## AUTHOR CONTRIBUTIONS

S.S. conceived the study, performed the analysis, and wrote the paper.

## Supporting information

FigS1

TableS1

## Data Availability

All data are available at the Sequence Read Archive under SRP075682 and PRJEB7673.
