# Supplementary material for: Transposable elements in individual genotypes of Drosophila simulans
Source: Ecol Evol. 2020 Mar 13;10(7):3402–12. doi: 10.1002/ece3.6134 (PMC7141027; doi:10.1002/ece3.6134)
Supplement: Supplementary file 1 — FigS1 [file ECE3-10-3402-s001.docx]

Supplementary Figure 1: Representative Tajima’s *D* from 2L of the African population of *D. simulans.*
